# Outer Membrane Vesicles Protect Gram-Negative Bacteria against Host Defense Peptides

**DOI:** 10.1128/mSphere.00523-21

**Published:** 2021-07-07

**Authors:** Melanie D. Balhuizen, Albert van Dijk, Jeroen W. A. Jansen, Chris H. A. van de Lest, Edwin J. A. Veldhuizen, Henk P. Haagsman

**Affiliations:** a Section of Molecular Host Defence, Division of Infectious Diseases and Immunology, Department of Biomolecular Health Sciences, Faculty of Veterinary Medicine, Utrecht University, Utrecht, The Netherlands; b Section of Cell Biology, Metabolism and Cancer, Division of Infectious Diseases and Immunology, Department of Biomolecular Health Sciences, Faculty of Veterinary Medicine, Utrecht University, Utrecht, The Netherlands; c Section of Immunology, Division of Infectious Diseases and Immunology, Department of Biomolecular Health Sciences, Faculty of Veterinary Medicine, Utrecht University, Utrecht, The Netherlands; University of Rochester

**Keywords:** Gram-negative bacteria, antibiotic resistance, antimicrobial peptides, host defense peptides, outer membrane vesicles

## Abstract

Host defense peptides (HDPs) are part of the innate immune system and constitute a first line of defense against invading pathogens. They possess antimicrobial activity against a broad spectrum of pathogens. However, pathogens have been known to adapt to hostile environments. Therefore, the bacterial response to treatment with HDPs was investigated. Previous observations suggested that sublethal concentrations of HDPs increase the release of outer membrane vesicles (OMVs) in Escherichia coli. First, the effects of sublethal treatment with HDPs CATH-2, PMAP-36, and LL-37 on OMV release of several Gram-negative bacteria were analyzed. Treatment with PMAP-36 and CATH-2 induced release of OMVs, but treatment with LL-37 did not. The OMVs were further characterized with respect to morphological properties. The HDP-induced OMVs often had disc-like shapes. The beneficial effect of bacterial OMV release was studied by determining the susceptibility of E. coli toward HDPs in the presence of OMVs. The minimal bactericidal concentration was increased in the presence of OMVs. It is concluded that OMV release is a means of bacteria to dispose of HDP-affected membrane. Furthermore, OMVs act as a decoy for HDPs and thereby protect the bacterium.

**IMPORTANCE** Antibiotic resistance is a pressing problem and estimated to be a leading cause of mortality by 2050. Antimicrobial peptides, also known as host defense peptides (HDPs), and HDP-derived antimicrobials have potent antimicrobial activity and high potential as alternatives to antibiotics due to low resistance development. Some resistance mechanisms have developed in bacteria, and complete understanding of bacterial defense against HDPs will aid their use in the clinic. This study provides insight into outer membrane vesicles (OMVs) as potential defense mechanisms against HDPs, which will allow anticipation of unforeseen resistance to HDPs in clinical use and possibly prevention of bacterial resistance by the means of OMVs.

## INTRODUCTION

When pathogens enter a host, they enter a hostile environment. Host species have developed many measures to eliminate and remove pathogens; however, pathogens have been evolving simultaneously. Well known is the development of antibiotic resistance, but pathogens also have found ingenious mechanisms to evade the host’s intrinsic immune system (1). One of the first innate defense molecules pathogens will encounter are host defense peptides (HDPs). HDPs are small, cationic molecules and have antibacterial activities against a broad range of pathogens. They are amphipathic, and this enables them to interact with bacterial membranes. Therefore, many HDPs are membrane active and exert their antibacterial function through membrane lysis (2).

One extensively studied HDP is LL-37. It is the only human cathelicidin, and much is known about its mechanism of action. LL-37 is an α-helical amphipathic peptide that was shown to interact with the bacterial membrane with its helical axis in a parallel fashion to the bacterial surface. It can form small, toroidal pores that cause cytoplasmic leakage but also provide opportunity for translocation of the peptide (3). LL-37 can bind to components of the peptidoglycan layer and interfere with its synthesis. Furthermore, LL-37 can interact with DNA and ribosomes and cause clustering of these components (4, 5). A second well-studied cathelicidin is the chicken cathelicidin CATH-2. It was shown to interact with lipopolysaccharide (LPS) and very rapidly localizes to the bacterial membrane, where it internalizes and, at higher concentrations, causes membrane permeabilization (4, 6). Another very active cathelicidin is porcine PMAP-36. It has a helical fold, similar to LL-37 and CATH-2, but differs from them by its ability to covalently dimerize, which enhances its pore-forming ability. Its mechanism of action is not fully understood, although it was shown to permeabilize bacterial membranes as well as cause clustering of intracellular targets, suggesting a multitarget mode of action (4, 7).

HDPs target multiple and vital parts of the bacterium, which makes it difficult to develop resistance. However, there are some bacteria that have developed mechanisms to counteract the antibacterial activity of HDPs (8–13). The most common mechanism is the secretion of molecules that render HDPs inactive. For example, the M1 protein of group A Streptococcus is able to confer protection against HDPs, even when expressed in other bacteria, by sequestering HDPs (14). PgtE from Salmonella not only interacts with α-helical antimicrobial peptides but was also shown to cleave these peptides (15). Furthermore, the secreted peptidylarginine deiminase (PAD) from Porphyromonas gingivalis is able to citrullinate peptides and thereby decrease the cationic charge, which is essential for the peptide’s function (16). An entire operon is upregulated in Clostridioides difficile to confer resistance against HDPs, of which the mechanism is not yet fully understood (17).

Since HDPs are membrane active molecules, bacteria also have been shown to alter their membranes to render HDPs inactive. Modification of phospholipids happens in multiple species through a conserved protein, MprF, which adds a lysine to phosphatidylglycerol and thereby neutralizes the negative charge (18). Furthermore, it has been shown that addition of external membrane, in the form of outer membrane vesicles (OMVs) (19), protects Escherichia coli against polymyxin B and colistin, two peptide antibiotics (20). Similarly, addition of OMVs protected Helicobacter pylori against LL-37 (21). In this work, we investigated bacterial defense against HDPs by exposing Gram-negative bacteria, E. coli, Bordetella bronchiseptica, and Pseudomonas aeruginosa, to sublethal concentrations of three HDPs: PMAP-36, CATH-2, and LL-37. OMV release was quantified, and the resulting OMVs were characterized. Furthermore, external OMVs were added to bacterial cultures to investigate whether this could increase resistance to HDPs. The results showed that CATH-2 and PMAP-36, but not LL-37, were able to induce OMV release. However, addition of OMVs to bacterial cultures showed protection against all three peptides.

## RESULTS

### CATH-2 and PMAP-36, but not LL-37, stimulate the release of OMVs in Gram-negative bacteria.

**FIG 1:**
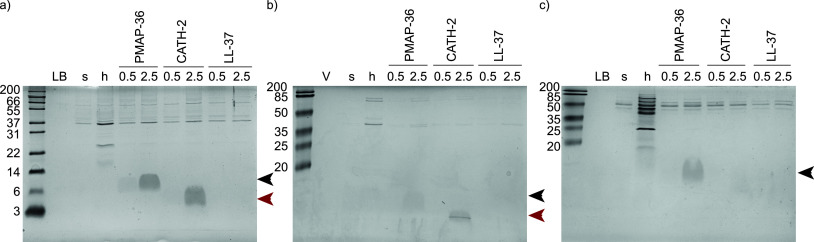
Coomassie-stained SDS-PAGE of isolated OMVs. OMVs were induced by HDPs and isolated from E. coli (a), B. bronchiseptica (b), and P. aeruginosa (c). Heat was applied as a stress control. LB/V, lysogeny broth/Verwey medium; s, sOMVs; h, hOMVs; 0.5, 0.5 μM; 2.5, 2.5 μM of the corresponding peptide. Black and red arrows point to PMAP-36 and CATH-2, respectively. Shown is a representative image of three experiments.

To investigate the effect of HDPs on OMV release by Gram-negative bacteria, we selected three bacterial species for our experiments, E. coli, B. bronchiseptica, and P. aeruginosa. Bacterial cultures were stimulated with two sublethal concentrations of different peptides, PMAP-36, CATH-2, and LL-37, and peptide-induced OMVs (pOMVs) were isolated. Heat treatment was applied as a control stressor, since it has been shown to induce OMV release, resulting in heat-induced OMVs (hOMVs) (22, 23). Isolated pOMVs and hOMVs were analyzed using Coomassie-stained SDS-PAGE and compared to spontaneous OMVs (sOMVs; Fig. 1). This confirmed that heat treatment indeed induced OMVs, as shown by an increase of protein band intensity. For the higher concentrations of PMAP-36 and CATH-2, a slight increase in protein band intensity was observed. As shown previously, PMAP-36 is present in the isolated pOMVs (Fig. 1A, black arrow) (23). CATH-2 is also present in the pOMV fraction (Fig. 1A, red arrow), but LL-37 is not. This indicates a difference in mechanism of action between the three peptides.

**FIG 2:**
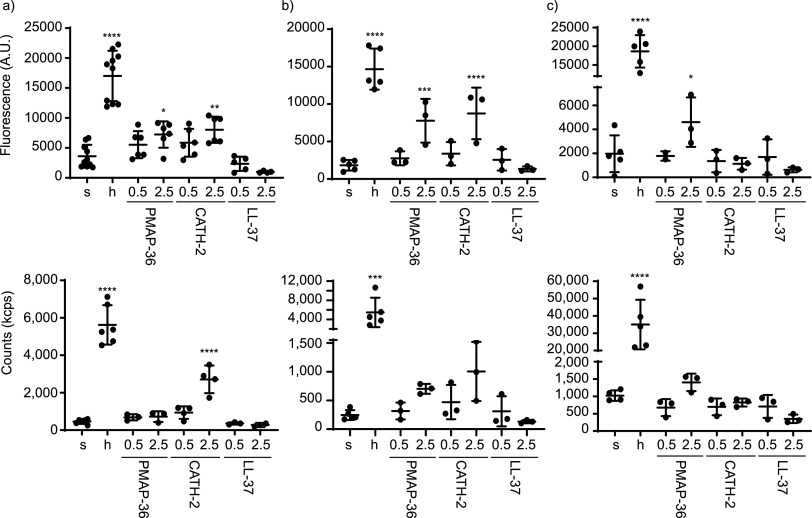
Quantification of isolated OMVs. OMVs were induced by HDPs and isolated from E. coli (a), B. bronchiseptica (b), and P. aeruginosa (c). (Top) FM4-64 lipid quantification of isolated OMVs. (Bottom) Particle count of isolated OMVs using DLS. Heat was applied as stress control. Results were corrected for medium signal. s, sOMVs; h, hOMVs; 0.5, 0.5 μM; 2.5, 2.5 μM of the corresponding peptide. Statistical analysis was performed using a linear mixed-model with *post hoc* Dunnett’s test (*n* = 3 to 9). *, *P* < 0.05; **, *P* < 0.01; ***, *P* < 0.001; ****, *P* < 0.0001.

To quantify differences in OMV release, first a bicinchoninic acid (BCA) assay was used (see Fig. S1 in the supplemental material). The high signal of medium alone interfered with accurate assessment of differences between treatments. However, heat treatment resulted in a large significant increase of OMV release by B. bronchiseptica and P. aeruginosa, as measured by the BCA assay. To quantify differences in OMV release based on lipids, the fluorescent FM4-64 membrane dye was used (Fig. 2, top). A significant increase in OMV release of all bacteria upon heat treatment was observed, as well as an increase in OMV release upon treatment with 2.5 μM PMAP-36 and CATH-2 for E. coli and B. bronchiseptica. However, for P. aeruginosa, only treatment with 2.5 μM PMAP-36 resulted in an increase of OMV release, indicating that the effect of CATH-2 is bacterium specific.

10.1128/mSphere.00523-21.1 FIG S1 BCA quantification of isolated OMVs. OMVs were induced by HDPs and isolated from E. coli (a), B. bronchiseptica (b), and P. aeruginosa (c). Medium was taken along in the isolation protocol as control. Heat was applied as stress control. LB/V, Lysogeny Broth/Verwey medium; s, sOMVs; h, hOMVs; 0.5, 0.5 μM; and 2.5, 2.5 μM of the corresponding peptide. Statistical analysis was performed using a linear mixed model with *post hoc* Dunnett’s test (*n* = 3 to 9). *, *P* < 0.05; ***, *P* < 0.001; ****, *P* < 0.0001. Download FIG S1, PDF file, 0.1 MB. Copyright © 2021 Balhuizen et al. 2021 Balhuizen et al. https://creativecommons.org/licenses/by/4.0/ This content is distributed under the terms of the Creative Commons Attribution 4.0 International license.

Since HDPs are membrane active and the intercalation of FM4-64 into the membrane might be influenced by the presence of peptides, an orthogonal technique was used to support the FM4-64 quantifications. Therefore, dynamic light scattering (DLS) was also used to estimate the number of OMV particles (Fig. 2, bottom). Since individual particles can be counted several times by this technique, it will not result in an absolute number, but relative outcomes can still be compared. The particle counts of the DLS overall corresponded to the results of the FM4-64 quantification, although in this assay not all differences reached statistical significance.

When comparing minimal bactericidal concentrations of the peptides used in this study, it was found that for B. bronchiseptica, minimal bactericidal concentration (MBCs) of PMAP-36 and CATH-2 were similar, 0.25 μM and 0.5 μM, respectively. For E. coli, MBCs of PMAP-36 and CATH-2 were 1.25 μM and 5 μM, respectively, only 4-fold different. However, for P. aeruginosa, the MBC of CATH-2 (20 μM) was 16-fold higher than the MBC of PMAP-36 (1.25 μM), possibly explaining the lack of OMV induction by CATH-2 for this bacterial species (Table 1). MBCs for LL-37 were consistently higher, possibly related to the lack of OMV induction by this peptide. Therefore, higher, but still sublethal, concentrations of LL-37 were tested for OMV induction of E. coli and P. aeruginosa (Fig. S2). Neither 5 nor 10 μM LL-37 was able to induce any OMVs for both species tested, suggesting that even at higher concentrations OMVs are not used as defense against LL-37.

**TABLE 1:** MBC values of PMAP-36, CATH-2 and LL-37 for E. coli, B. bronchiseptica, and P. aeruginosa^*a*^

Compound	E. coli ATCC 25922	B. bronchiseptica	P. aeruginosa PAO1
PMAP-36	1.25	0.25	1.25
CATH-2	5	0.5	20
LL-37	10	1.25	>40

a Concentrations were determined with track dilution assays and expressed in micromolar. Values for E. coli and P. aeruginosa were determined in LB. Values for B. bronchiseptica are in Verwey medium.

10.1128/mSphere.00523-21.2 FIG S2 Quantification of E. coli and P. aeruginosa OMVs induced by 5 and 10 μM LL-37. OMVs were induced by LL-37 and isolated from E. coli and P. aeruginosa. Left, BCA quantification; right, FM4-64 lipid quantification of isolated OMVs. Results were corrected for medium signal. Download FIG S2, TIF file, 6.4 MB. Copyright © 2021 Balhuizen et al. 2021 Balhuizen et al. https://creativecommons.org/licenses/by/4.0/ This content is distributed under the terms of the Creative Commons Attribution 4.0 International license.

### CATH-2 and PMAP-36, but not LL-37, are present in the isolated OMVs.

**FIG 3:**
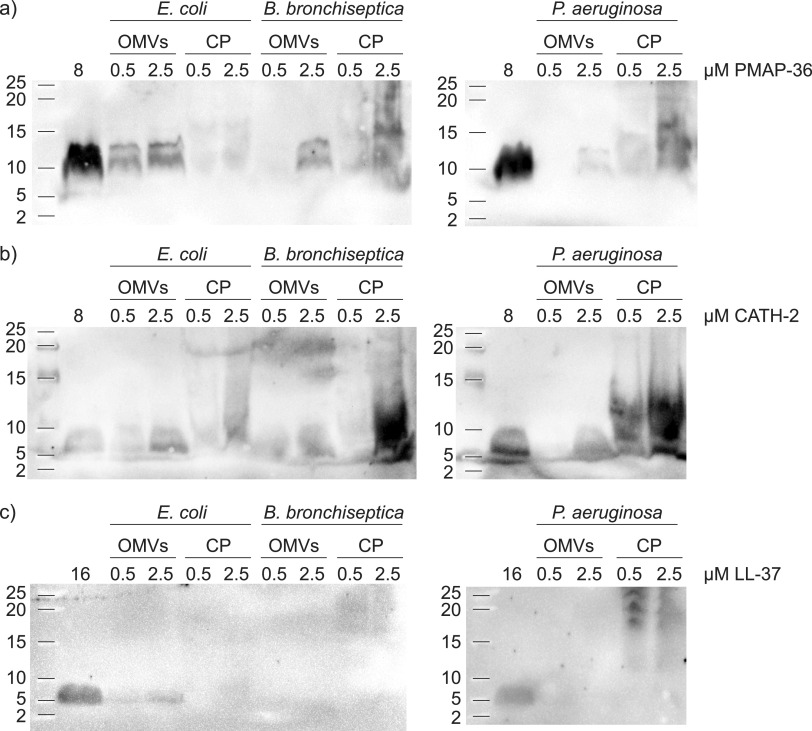
Western blot analysis of isolated OMVs and bacterial cell pellet (CP), stained for PMAP-36 (a), CATH-2 (b), and LL-37 (c). OMVs were induced by two concentrations of HDPs (0.5 and 2.5 μM) and isolated from E. coli, B. bronchiseptica, and P. aeruginosa, and bacterial cell pellets were collected during isolation. Equal parts of the total amount of OMV and CP fraction were loaded to compare HDP presence. Synthetic peptide (8 μM for PMAP-36 and CATH-2, 16 μM for LL-37) was loaded as a positive control.

To confirm the presence of HDPs in the OMVs, these were investigated by Western blotting and stained with the corresponding antibody (Fig. 3). This indeed confirmed that the low-molecular-weight patches observed before on Coomassie-stained SDS-PAGE corresponded to PMAP-36 and CATH-2. No LL-37 was detected in the OMV fraction, which is in line with the absence of peptide on Coomassie-stained SDS-PAGE. If bacteria utilize OMV release as a means to dispose of HDP-affected membrane, one would expect that OMVs would be enriched in HDPs compared to bacterial membranes. Therefore, the presence of HDPs was also investigated in the bacterial pellet, separated from the OMVs with centrifugation, after HDP treatment. To analyze this, equal parts of the total amount of the bacterial pellet and isolated OMV fraction were loaded. This allows for comparison between bacterial cell pellet and OMV fraction of corresponding samples. Analysis between corresponding samples showed that PMAP-36 is preferentially found in the OMV fraction for E. coli, roughly equally distributed between OMVs and bacterial pellet for B. bronchiseptica and preferentially found in the bacterial pellet for P. aeruginosa (Fig. 3a). CATH-2 was found equally in the bacterial pellet and OMV fraction for E. coli but preferentially in the bacterial pellet for B. bronchiseptica and P. aeruginosa (Fig. 3b). This already shows differences between the two peptides. Remarkably, LL-37 was found in the OMV fraction of E. coli but not in the bacterial pellet. It was not detected in the bacterial pellet or in the OMV fraction of B. bronchiseptica and P. aeruginosa (Fig. 3c). This indicates that the peptide mainly resided in the OMV supernatant after ultracentrifugation, but this showed no peptide either (data not shown). These results clearly suggest that bacterial species have different defense mechanisms toward HDPs.

### HDP induced OMVs differ in morphology.

**FIG 4:**
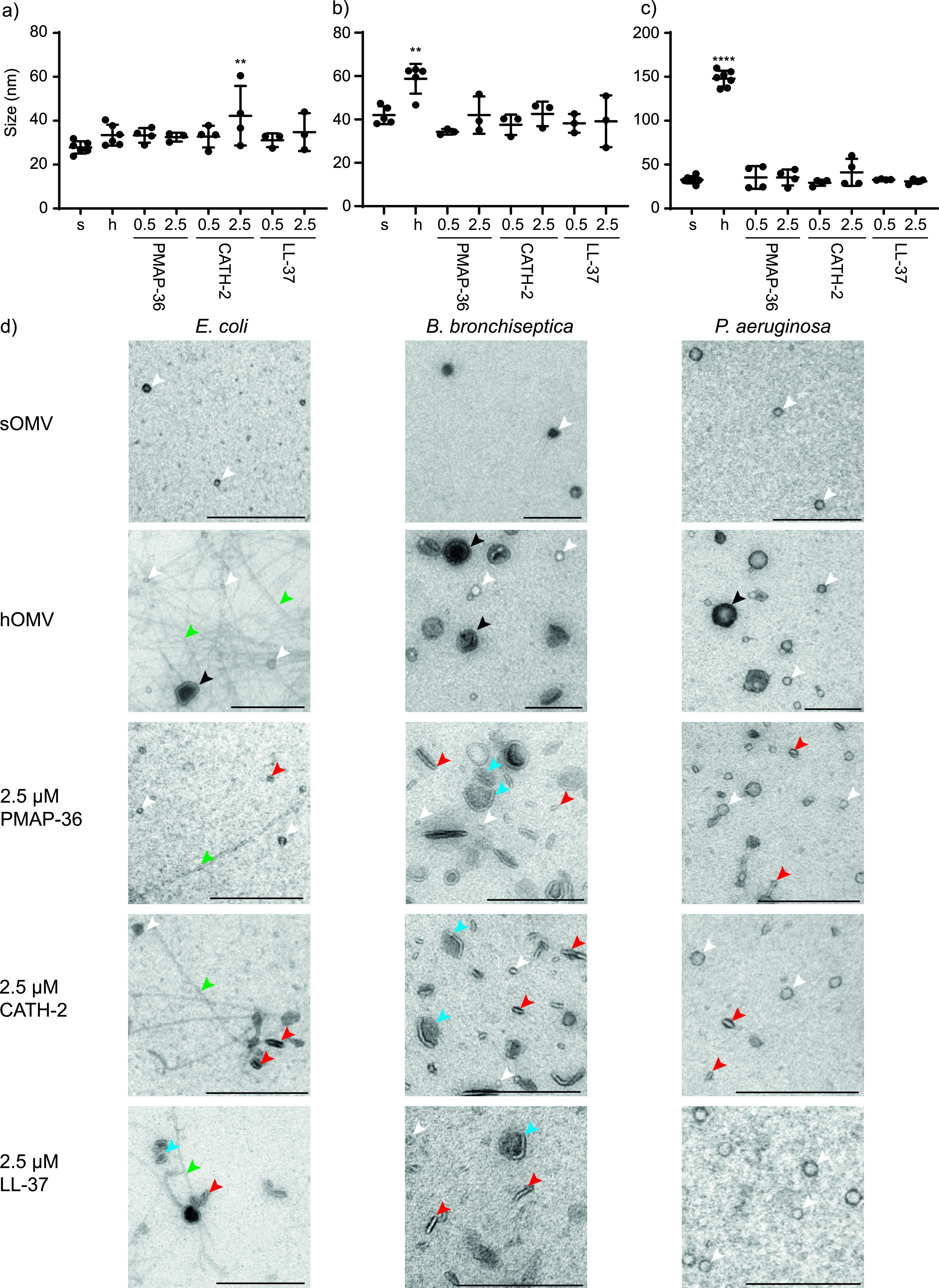
Morphological properties of isolated OMVs. OMVs were induced by HDPs and isolated from E. coli (a), B. bronchiseptica (b), and P. aeruginosa (c). Heat was applied as stress control. (a to c) Size determination by DLS. s, sOMVs; h, hOMVs; 0.5, 0.5 μM; 2.5, 2.5 μM the corresponding peptide. Statistical analysis was performed using a linear mixed model with *post hoc* Dunnett’s test (*n* = 3 to 6). **, *P* < 0.01; ****, *P* < 0.0001. (d) Electron microscopy. White arrows point to vesicles with the morphology of sOMVs, and black arrows to larger hOMVs. Red arrows show disc-like pOMVs, and blue arrows show pOMVs that have split open and still have cargo associated. Green arrows show flagella. Scale bars represent 200 nm. Shown is a representative image of three experiments.

The effect of HDP treatment of bacteria on the resulting pOMV size was determined using DLS. This revealed that spontaneous as well as HDP-induced OMVs had a diameter of approximately 30 to 40 nm for all three Gram-negative bacteria (Fig. 4a to c). However, heat treatment affected the size of resulting hOMVs but not to the same extent for all bacteria. For B. bronchiseptica, hOMVs of 60 nm were measured, while hOMVs of E. coli were similar in size to sOMVs. However, a very large effect was observed for P. aeruginosa, where hOMVs were found to have an average diameter of 150 nm.

In order to assess morphology and integrity, OMVs were visualized using electron microscopy (EM) (Fig. 4d, Fig. S3). This revealed that OMVs were indeed quite small, in concordance with the DLS results (Fig. 4d, white arrows). It also showed that heat treatment affects OMV appearance, with larger and darker OMVs present (Fig. 4d, black arrows). Peptide treatment did not affect OMV size but did affect morphology, as observed by EM. OMVs induced by all three HDPs revealed disc-like shapes (Fig. 4d, red arrows). The association of cargo, represented by darker patches along these disc-like OMVs, suggests that the OMVs split open after release from the bacterium (Fig. 4d, blue arrows). Remarkably, differences in OMV morphology were observed for E. coli upon treatment, and flagella were observed (Fig. 4d, green arrows). These were mostly observed after heat treatment of E. coli but also after peptide treatment. For B. bronchiseptica and P. aeruginosa, flagella were occasionally observed after heat treatment but not to the same extent as that for E. coli (data not shown). For P. aeruginosa, smaller fragments of flagella were also observed, possibly interfering with the DLS size measurement and explaining the large increase in diameter upon heat treatment.

10.1128/mSphere.00523-21.3 FIG S3 Morphology of OMVs resulting from bacteria treated with 0.5 μM peptide. OMVs were induced by 0.5 μM the respective HDP and isolated from E. coli, B. bronchiseptica, and P. aeruginosa. Medium shows the corresponding growth medium of the respective bacterium. White arrows point to sOMVs. Red arrows show disc-like pOMVs and blue arrows show pOMVs that have split open and still have cargo associated. Green arrows show flagella. Scale bars represent 200 nm. Shown is a representative image of three experiments. Download FIG S3, TIF file, 2 MB. Copyright © 2021 Balhuizen et al. 2021 Balhuizen et al. https://creativecommons.org/licenses/by/4.0/ This content is distributed under the terms of the Creative Commons Attribution 4.0 International license.

**FIG 5:**
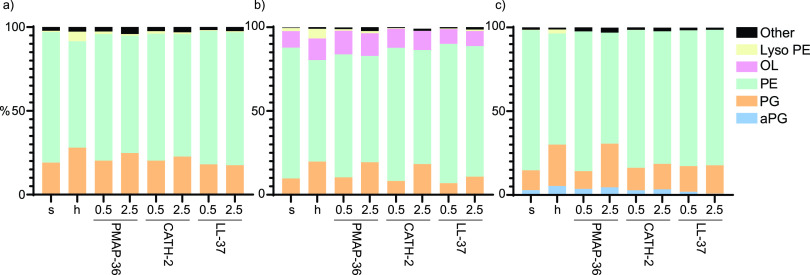
Lipidomic analysis of isolated OMVs from E. coli (a), B. bronchiseptica (b), and P. aeruginosa (c) induced by no treatment (s), heat treatment (h), or different concentrations (0.5 or 2.5 μM) of indicated HDPs. The main OMV components are phosphatidylethanolamine (PE) and phosphatidylglycerol (PG). Ornithine lipid (OL) was only found in B. bronchiseptica. aPG, acyl-phosphatidylglycerol. Other includes phosphatidic acid (PA), phosphatidylserine (PS), dilyso-cardiolipin (DLCL), and lipid groups at <2%.

To further investigate OMV characteristics, lipidomic analysis was performed using mass spectrometry. In this analysis, not only phospholipids but also ornithine lipids were measured for B. bronchiseptica, as these were described before to be present in OMVs (23). OMVs of all three different bacterial species consisted mainly of phosphatidylethanolamine (PE) and phosphatidylglycerol (PG) (Fig. 5). Slight differences between species were observed, such as relatively more acyl-phosphatidylglycerol (aPG) in P. aeruginosa than E. coli and B. bronchiseptica (Table S1).

10.1128/mSphere.00523-21.5 Table S1 Percentages of lipid species observed in isolated OMVs. OMVs were isolated from E. coli, B. bronchiseptica, and P. aeruginosa, induced by no treatment (sOMV), heat treatment (hOMVs), or different concentrations of the indicated HDPs. aPG, acyl-phosphatidylglycerol; DLCL, dilyso-cardiolipin; lyso PE, lyso-phosphatidylethanolamine; OL, ornithine lipid; PA, phosphatidic acid; PE, phosphatidylethanolamine; PG, phosphatidylglycerol; PS, phosphatidylserine. Download Table S1, DOCX file, 0.02 MB. Copyright © 2021 Balhuizen et al. 2021 Balhuizen et al. https://creativecommons.org/licenses/by/4.0/ This content is distributed under the terms of the Creative Commons Attribution 4.0 International license.

When peptide- and heat-induced OMVs were compared to spontaneous OMVs, several differences in lipid composition were observed. A relative increase in negatively charged PG occurred in OMVs induced with 2.5 μM PMAP-36 and 2.5 μM CATH-2 but not 2.5 μM LL-37, supporting the observation that LL-37 does not induce OMV release (Fig. 5). Furthermore, OMVs from P. aeruginosa induced with 2.5 μM CATH-2 do not display an increase in PG lipids, confirming earlier results where CATH-2 was unable to induce P. aeruginosa OMVs (Fig. 5c). A relative increase of lysophospholipids was found in OMVs of all three bacterial species upon heat treatment (23).

### OMVs protect bacteria from HDPs.

To investigate whether the release of OMVs in response to peptide treatment is indeed a means of the bacterium to defend itself, track dilution assays were performed. Two sets of E. coli hypervesiculating mutants were investigated, one with a deletion of the outer membrane protein OmpA and the other with a deletion of the lipoprotein Lpp, both important for outer membrane tethering to either the peptidoglycan or inner membrane. These genetically modified bacteria have an increased production of OMVs (24, 25), which might protect them against HDPs, but the deletion may also influence membrane stability of the bacterium, which should be taken into consideration. Therefore, the supernatant of the hypervesiculating mutants and wild-type bacteria was used to investigate protective capabilities against HDP killing of the wild-type bacteria to eliminate bacterial differences. This revealed a protective effect against CATH-2, PMAP-36, and LL-37 (Fig. S4).

10.1128/mSphere.00523-21.4 FIG S4 MBC comparisons of two E. coli strains and corresponding hypervesiculating mutant. Track dilution assays were performed for two E. coli strains and corresponding hypervesiculating mutant using three different peptides. Shown is the mean with SEM (*n* = 3). Statistical analysis was performed using a two-way ANOVA with *post hoc* Sidak between corresponding mutant and wild-type for each concentration. *, *P* < 0.05; ***, *P* < 0.001; ****, *P* < 0.0001. Download FIG S4, PDF file, 0.8 MB. Copyright © 2021 Balhuizen et al. 2021 Balhuizen et al. https://creativecommons.org/licenses/by/4.0/ This content is distributed under the terms of the Creative Commons Attribution 4.0 International license.

**FIG 6:**
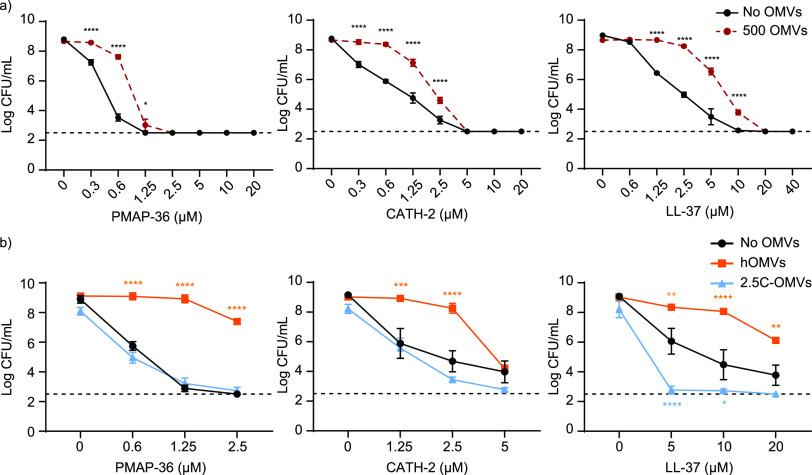
Assessment of killing of E. coli by HDPs with and without the addition of external (a) sOMVs, (b) hOMVs, or OMVs induced by 2.5 μM CATH-2 (2.5C-OMVs). OMVs from E. coli were isolated and 500 AU were added to the bacterium-HDP mixture during incubation. In the control, an equal volume of Tris buffer was added to the bacteria-HDP mixture. (a) Shown is the mean from four independent experiments with standard errors of the means (SEM). Statistical analysis was performed for each peptide concentration using a two-way analysis of variance with *post hoc* Sidak. *, *P* < 0.05; ****, *P* < 0.0001. (b) Shown is the mean from three independent experiments with SEM. Statistical analysis was performed compared to the control without OMVs added using a two-way ANOVA with *post hoc* Dunnet. *, *P* < 0.05; **, *P* < 0.01; ***, *P* < 0.001; ****, *P* < 0.0001.

Since the supernatant contains several components, isolated E. coli sOMVs also were added during track dilution assays to investigate the potential protective effect of OMVs against the antibacterial activity of HDPs. The concentration of sOMVs added in these track dilution assays was equated with the amount of sOMVs isolated after 2 h of logarithmic growth of E. coli, being 500 arbitrary units (AU) as defined using the FM4-64 lipid dye. Addition of isolated sOMVs during the incubation of HDPs with bacteria in the track dilution assay resulted in a protective effect of sOMVs against CATH-2, PMAP-36, and LL-37 killing (Fig. 6a). This suggests that the mere presence of outer membrane vesicles can act as a decoy for HDPs. Additionally, the protective effects of hOMVs and OMVs induced by 2.5 μM CATH-2 (2.5C-OMVs) were investigated, since these conditions significantly increased OMV release. This revealed that hOMVs also protected E. coli against killing by all three peptides even more than sOMVs for PMAP-36. However, 2.5C-OMVs did not protect E. coli and even enhanced killing by LL-37 (Fig. 6b).

## DISCUSSION

In this study, the Gram-negative bacteria E. coli, B. bronchiseptica, and P. aeruginosa were exposed to sublethal concentrations of the HDPs PMAP-36, CATH-2, and LL-37, and OMV release was studied to determine the role of OMVs as a defense mechanism against these HDPs. Furthermore, we assessed whether an increase in OMVs in the culture medium could protect E. coli from HDP killing. Treatment with either PMAP-36 or CATH-2 induced OMV release, but treatment with LL-37 did not have this effect (Fig. 2). Remarkably, CATH-2 was only effective for E. coli and a clinical isolate of B. bronchiseptica but not P. aeruginosa. It would be interesting to investigate the OMV induction by HDPs further for more clinically relevant bacterial species and strains. Experiments were performed in rich media to exclude other environmental factors affecting OMV release, and observed effects were solely caused by heat or HDP treatment.

Peptide-dependent differences in OMV release may be partially explained by their different antimicrobial mechanisms. For PMAP-36 and CATH-2, it was shown that they both interact strongly with LPS of E. coli in a biphasic manner. LL-37 only interacts weakly with LPS in a monophasic manner (4). Furthermore, electron micrographs showed clustered DNA and ribosomes for LL-37, demonstrating an intracellular-active mechanism, while CATH-2 localized intracellularly at sublethal concentrations but disrupted membranes at lethal concentrations, demonstrating a membrane-active mechanism (4, 6). PMAP-36 was shown to disrupt membranes (7, 26) but also showed clustered DNA and ribosomes in electron microscopic analysis of E. coli (4), suggesting a combination of membrane and intracellular targets for PMAP-36. This shows that the three HDPs have different antibacterial mechanisms of action and may explain why LL-37 does not induce OMV release, even at higher concentrations. Since LL-37 targets intracellular processes and is not localized to the membrane, the bacterium does not require the disposal of membrane in the form of an OMV. This suggests that OMV release is a means of the bacterium to dispose of membrane affected by peptide.

Western blot analysis of bacterial pellets showed PMAP-36 and CATH-2 present in both the OMV fraction and the bacterial cell pellet, but LL-37 was not present in most samples, except for the OMV pellet of E. coli (Fig. 3). The distribution was different for each bacterium, where P. aeruginosa and B. bronchiseptica both contained mostly peptide in the bacterial cell pellet and E. coli contained mostly peptide in the OMVs. This suggests that PMAP-36 and CATH-2 can be neutralized by incorporation into OMVs but that this defense mechanism differs between bacterial species. Perhaps some species rely more on other defense mechanisms instead of elimination of HDPs by OMVs. LL-37 could barely be detected in the bacterial cell pellet, OMV fraction, or supernatant, suggesting either concentrations are too low to be detected on Western blot, for example, by breakdown of the peptide, or the antibody is not powerful enough. Only a low signal was detected in the OMVs of E. coli induced with 2.5 μM LL-37. The positive control for LL-37 had to be doubled in concentration, being 16 μM instead of 8 μM, to be properly detected by the antibody used, suggesting a higher detection limit. Therefore, Western blot detection of LL-37 with this antibody might not be conclusive.

A difference was also observed in the bacterial response to CATH-2 specifically. When MBC values were investigated, it was found that the MBC of CATH-2 for P. aeruginosa was 16-fold higher than that of PMAP-36, while for E. coli and B. bronchiseptica MBCs for CATH-2 and PMAP-36 were only 2- or 4-fold different. Perhaps CATH-2 fails to induce OMVs in P. aeruginosa simply because the concentration used is too low. A similar mechanism could be at play for LL-37.

Still, the question remains why the MBC of CATH-2 is so much higher for P. aeruginosa than for E. coli and B. bronchiseptica. The first molecule CATH-2 will encounter in all of these bacteria is LPS, which differs per bacterium. B. bronchiseptica LPS is 90% penta-acylated and 10% hexa-acylated, with glucosamine groups attached to the phosphates (27). Similarly, P. aeruginosa PAO1 LPS molecules are 75% penta-acylated and 25% hexa-acylated (28). On the other hand, E. coli ATCC 25922 LPS is fully hexa-acylated (29, 30). All LPS structures contain an O-antigen (31–34) and are comparable in lipid A composition, but perhaps the differences in the core and O-antigen will influence binding by CATH-2 and its effectiveness. This could be investigated in the future by affinity studies.

When OMV characteristics were assessed, it was shown that heat treatment significantly influenced OMV size for B. bronchiseptica and P. aeruginosa (Fig. 4b and c). It was also observed that heat treatment influenced the lipid classes present in the hOMVs, which was already described for B. bronchiseptica (23). An increase in lysophospholipids was observed in OMVs of all species (Fig. 5), although it was less pronounced in P. aeruginosa. This could be due to the lack of outer membrane phospholipase A (*pldA*) in P. aeruginosa, which was implicated in B. bronchiseptica to cause the increase of lysophospholipids in hOMVs (23). Protein BLAST searches using the sequence of E. coli *pldA* as a query did not reveal the presence of a *pldA* homolog in P. aeruginosa PAO1. These lysophospholipids contain only one fatty acid tail and, therefore, induce a positive curvature in the membrane (35).

The presence of many flagellum-like structures was observed in the electron microscopic graphs of isolated OMVs. In literature, P. aeruginosa is described as monotrichous, while E. coli and B. bronchiseptica are described as peritrichous (36, 37). However, *Bordetellae* flagellar synthesis is regulated by the Bvg regulon and is decreased at growth temperatures of 37°C and above (38). This was consistent with the number of flagellar structures observed, where the most were observed for E. coli (Fig. 4d, green arrows). It was shown that this elevated temperature alters the OMV composition by an increase of lysophospholipids (Fig. 5) and thereby may also affect bacterial membrane fluidity and proper attachment of flagella. These flagella are connected to the outer membrane by the L-ring, and placement is thought to be regulated by marker proteins that interact with phospholipids (36). However, the effect of phospholipid composition on flagellar connection is currently unknown. For E. coli, flagella were also observed in samples treated with higher peptide concentrations. HDPs could disrupt membrane integrity in the case of PMAP-36 or CATH-2 or perhaps interfere with translation of flagellar protein components in the case of LL-37 (4) and thereby prevent proper flagellar attachment or cause flagella to detach.

Furthermore, the lipidome and morphology of peptide-induced OMVs were altered. pOMVs obtained a disc-like morphology, which was especially pronounced for B. bronchiseptica (Fig. 4d, red arrows). Additionally, OMVs induced with 2.5 μM PMAP-36 or CATH-2 showed a relative increase in negatively charged PG lipids, possibly due to a preferred interaction with the positively charged peptides (Fig. 5). In accordance with the quantifications, this effect was not observed for 2.5 μM CATH-2 in P. aeruginosa. However, an increase in PG lipids would not explain the observed disc-like morphology of pOMVs, indicating that other mechanisms are at play.

The association of darker patches along these disc-like pOMVs suggest they have split open after formation and still have cargo associated (Fig. 4d, blue arrows). However, not all disc-like pOMVs have darker patches associated with them, which indicates that these discs have been poked out of the outer membrane of the bacterium directly. This would resemble the mechanism described for nanodisc formation. Nanodiscs are small phospholipid bilayer discs encircled by an amphipathic scaffold protein (39). HDPs are amphipathic peptides and could function as nanodisc scaffold proteins. Natural lipoproteins, like apolipoprotein J, involved in lipid and cholesterol transport were previously shown to be able to form nanodiscs (40). A different explanation for the presence of these disc-like pOMVs could be that pOMVs have been compressed during sample preparation due to decreased stability because of the HDPs present in the pOMVs. Whether this is a true phenomenon or a sample preparation artifact has to be investigated using orthogonal imaging techniques, but this will be difficult due to the small size of these OMVs. However, when 4  μM PMAP-36 was added to isolated B. bronchiseptica sOMVs, this did not lead to altered morphology or decreased stability (data not shown), suggesting the phenomenon occurs during bacterial stimulation.

When assessing the protective effect of OMVs against the bactericidal action of HDPs, two sets of different hypervesiculating mutants were utilized. Initially these were tested in HDP killing assays, but no differences were observed (data not shown). This was presumably due to decreased membrane stability in the mutant counteracting the protective effect of increased OMV release. Therefore, the assays were performed using wild-type bacteria diluted in supernatant containing sOMVs of wild-type or hypervesiculating mutant E. coli. This indeed showed a protective effect of the sOMVs in the supernatant of the hypervesiculating mutants (see Fig. S3 in the supplemental material). Albeit not quantified, SDS-PAGE did suggest that deletion of *lpp* had a larger increase in OMV formation than deletion of o*mpA* (data not shown). This can explain the larger protective effect observed in the *Δlpp* mutant compared to the *ΔompA* mutant (Fig. S3).

Addition of isolated sOMVs or hOMVs also protected E. coli from killing by PMAP-36, CATH-2, and LL-37. However, 2.5C-OMVs did not protect E. coli and even enhanced killing by LL-37. Potentially this is caused by pore formation due to the CATH-2 that is present in the OMVs and apparently still active, which enables easier access for LL-37. Interestingly, addition of sOMVs protected the bacteria against LL-37 (Fig. 6a), but bacteria did not produce OMVs in response to LL-37 even at higher concentrations (Fig. 2, Fig. S2). The rationale suggests that since LL-37 targets intracellular processes, the bacterium does not need to dispose of large quantities of membrane since that is not where most of the peptide is localized. However, some LL-37 was detected in OMVs of E. coli induced with 2.5 μM LL-37 (Fig. 3). Perhaps smaller amounts of OMVs can be sufficient to dispose of all the LL-37 in the membrane. However, to reach the intracellular target, LL-37 needs to traverse through the membrane, possibly by interacting and diffusing through it, since it was shown to interact with membranes (3). Therefore, the addition of external membranes, in the form of OMVs, might slow the entry of LL-37 into bacteria, since it will interact with both OMVs and bacterial membranes. A similar principle of protection by OMV was observed for bacteriophages, where OMVs were shown by EM to interact with these bacteriophages and thereby protect the bacterial culture against killing by these viruses (20). OMVs can not only act as a physical barrier but perhaps also contain proteases or other factors that decrease HDP function and enhance bacterial survival. Membrane vesicles from Streptococcus suis, for instance, were shown to contain a serine protease (41). However, in the OMVs induced with HDPs, intact peptide was observed on Western blotting (Fig. 3), suggesting the peptide-induced OMVs of B. bronchiseptica do not contain proteases.

Altogether, these data show OMVs as a possible defense mechanism against membrane-active antibacterial compounds. We hypothesize that bacteria try to dispose of membranes affected by membrane-active antibacterial compounds in the form of an OMV. This mechanism was even found in a clinically isolated B. bronchiseptica, suggesting this process is relevant *in vivo.* This does suggest that an antivesiculation drug will increase effectiveness of membrane-active antibacterial compounds. Furthermore, previous studies have shown that nonvesiculating mutants are often lethal, suggesting that OMV formation is an essential process for bacteria (42) and an interesting drug target.

## MATERIALS AND METHODS

### Peptide synthesis.

PMAP-36 and CATH-2 were synthesized by Fmoc chemistry at China Peptides (CPC Scientific, Sunnyvale, CA, USA). LL-37 was synthesized by Fmoc-chemistry at the Academic Centre for Dentistry Amsterdam (Amsterdam, the Netherlands). All peptides were purified to a purity of >95% by reverse-phase high-performance liquid chromatography. Sequences and characteristics of the peptides are shown in Table 2.

**TABLE 2:** Sequence, length, and charge of studied peptides

Peptide	Sequence	Length	Charge
LL-37	LLGDFFRKSKEKIGKEFKRIVQRIKDFLRNLVPRTES	37	6+
CATH-2	RFGRFLRKIRRFRPKVTITIQGSARF-NH_2_	26	8+
PMAP-36	Ac-GRFRRLRKKTRKRLKKIGKVLKWIPPIVGSIPLGCG	36	13+

### Bacterial species and growth conditions.

E. coli ATCC 25922, a clinical isolate of B. bronchiseptica from pig (BB-P19; Veterinary Microbiological Diagnostic Center [VMDC], Utrecht University) and the laboratory strain P. aeruginosa PAO1 were used throughout this study. Both E. coli and P. aeruginosa were grown on tryptone soy agar (TSA) plates (Oxoid Ltd., Basingstoke, Hampshire, UK). Liquid cultures were grown in lysogeny broth (LB) containing 1% yeast extract (Becton, Dickinson and Company, Sparks, USA), 1% NaCl (Merck, Darmstadt, Germany), and 0.5% tryptone (Becton, Dickinson and Company). B. bronchiseptica was grown on Difco Bordet-Gengou (BG) agar plates (Becton, Dickinson and Company) containing 1% glycerol (Merck) supplemented with 15% (vol/vol) defibrinated sheep blood (Oxoid Ltd.). Liquid cultures were grown in Verwey medium (pH 7.4) (43) containing 0.1% (wt/vol) starch from potato (S2004; Sigma-Aldrich, St. Louis, MO, USA), 0.02% (wt/vol) KCl, 0.05% (wt/vol) KH_2_PO_4_, 0.01% (wt/vol) MgCl_2_·6 H_2_O (all from Merck), 0.002% (wt/vol) nicotinic acid (Sigma-Aldrich), 1.4% (wt/vol) Bacto Casamino Acids (Becton, Dickinson and Company), and 0.001% (wt/vol) l-glutathione reduced (Sigma-Aldrich).

### OMV isolation.

OMVs were isolated as described before (23). In short, bacteria were grown overnight to an optical density of approximately 1.5. Before OMV isolation was initiated, bacteria were treated for 1 h. Subsequently, bacterial cells were removed by centrifugation for 30 min at 4,700 × *g*. The supernatant was passed through a 0.45-μm Whatman filter (GE Healthcare, Chicago, IL, USA) and centrifuged at 40,000 rpm for 2 h at 4°C (Ti-70 rotor; Beckman Coulter, Brea, CA, USA). The supernatant was decanted, and the transparent pellet was dissolved in 2 mM Tris (pH 7.5; Sigma-Aldrich) in a volume corresponding to 2% of the bacterial culture.

### Generation of PMAP-36 antibody.

Rabbit polyclonal antibody against synthetic PMAP-36 peptide (GRFRRLRKKTRKRLKKIGKVLKWIPPIVGSIPLGCG-amide) was generated at Biogenes (Berlin, Germany). Twenty-five micrograms of PMAP-36 peptide was synthesized by Fmoc chemistry at a purity of >80% with quality control by high-performance liquid chromatography (HPLC) and mass spectrometry. Five milligrams of PMAP-36 was conjugated to limulus polyphemus hemocyanin (LPH) with 4-(N-maleimidomethyl)-cyclohexane-1-carboxylic acid N-hydroxysuccinimide ester and used to immunize two rabbits. Immunization followed a schedule with several boosts during several months and enzyme-linked immunosorbent assay (ELISA) titer testing of antisera, after which antisera from both rabbits were collected after final bleeding. Hundreds of milliliters of pooled antiserum was purified by affinity chromatography on a PMAP-36 coated CNBr-Sepharose column. Monospecific IgG was then eluted from the column with 0.2 M glycine–HCl buffer containing 250 mM NaCl (pH 2.2), neutralized with 2 M Tris-HCl (pH 7.5) and filtered (pore width, 0.45 μm) to remove any remaining debris. To conserve the antibody, 0.1% ProClin 300 was added.

### SDS-PAGE.

Acrylamide gels (14%) were prepared as previously described (44). For Coomassie staining, OMVs were diluted in 2× sample buffer containing 5%, vol/vol, β-mercaptoethanol (Sigma-Aldrich) and boiled for 10 min at 95°C, and 20 μl was loaded on the gel. Gels were run for 30 min at 50 V and then another 60 min at 150 V. Gels were stained with 0.1% (wt/vol) Coomassie brilliant blue R-250 (Serva, Heidelberg, Germany) in 50:40:10 MilliQ (MQ)-methanol-acetic acid (Sigma-Aldrich, Honeywell, Charlotte, NC, USA) and destained overnight in 80:10:10 MQ-methanol-acetic acid. For Western blots, gels were transferred to activated nitrocellulose membranes (Bio-Rad, Hercules, CA, USA) using Transblot Turbo (Bio-Rad) according to the manufacturer’s protocol. Membranes were blocked for 1 h with 5% bovine serum albumin (BSA; Sigma-Aldrich) in phosphate-buffered saline (PBS; Thermo Fisher Scientific) at room temperature (RT) and washed three times with TBS-T (0.9 M Tris, 25 M NaCl [both Merck], and 0.1, vol/vol%, Tween 20 [Serva]). Primary antibodies were diluted 1:2,500 for CATH-2 (45) and PMAP-36 and 1:1,000 for LL-37 (Phoenix Pharmaceuticals, CA, USA) in 1% BSA in PBS, and blots were incubated overnight at 4°C. After three washes with TBS-T, blots were incubated with goat anti-rabbit peroxidase antibodies, diluted 1:5,000, for 1 h at RT. Blots were washed again three times with TBS-T and once with PBS and developed using the clarity Western ECL substrate kit (Bio-Rad) according to the manufacturer’s protocol. Gels and blots were imaged with a Universal Hood III (Bio-Rad).

### BCA assay.

Total protein concentration of isolated OMVs was determined using the Pierce BCA assay (Thermo Fisher Scientific). In short, 25 μl of sample, supplemented with 2% SDS (Invitrogen, Carlsbad, CA, USA), was incubated with 200 μl of working reagent at 37°C for 2 h. Absorbance was measured at 562 nm with the FLUOstar omega (BMG Labtech, Ortenberg, Germany). BSA was used as the reference.

### FM4-64 assay.

Total lipid concentration of isolated OMVs was determined using the membrane-inserting fluorescent dye FM4-64 (Invitrogen). Samples (25 μl) were incubated with 200 μl FM4-64 (2.25 μg/ml) at 37°C for 10 min. Samples were excited at 485 nm and fluorescence was measured at 670 nm with the FLUOstar omega.

### DLS.

Samples for DLS were diluted 10-fold in 2 mM Tris. Samples were measured in microvolume cuvettes (Sarstedt, Nümbrecht, Germany) on a Zetasizer nano (Malvern Panalytical, Malvern, UK) with a scatter angle of 173°. The standard polystyrene latex was used with a refractive index of 1.590 and absorbance of 0.010. Water was used as the solvent (viscosity of 0.8872, refractive index of 1.330). Three measurements of 10 to 20 samplings were performed at 25°C.

### Lipidomics.

OMV pellets were obtained as described above. Lipids from OMVs were extracted using the method described by Bligh and Dyer (46). Lipid extracts were dried under N_2_, dissolved in 100 μl of chloroform and methanol (1:1), and injected (10 μl) into a hydrophilic interaction liquid chromatography column (2.6-μm HILIC; 100 Å, 50 by 4.6 mm; Phenomenex, CA). Lipid classes were separated by gradient elution on an Infinity II 1290 ultrahigh-performance liquid chromatograph (Agilent, CA) at a flow rate of 1 ml/min. A mixture of acetonitrile and acetone (9:1, vol/vol) was used as solvent A, while solvent B consisted of a mixture of acetonitrile, MQ (7:3, vol/vol) with 50 mM ammonium formate. Both A and B contained 0.1% (vol/vol) formic acid. Gradient elution was done with the following format (time in minutes, percent % B): (0, 0), (1, 3, 50), (3.01, 100), (4, 100). No reequilibration of the column was necessary between successive samples. The column effluent was connected to a heated electrospray ionization source of an Orbitrap Fusion mass spectrometer (Thermo Scientific, MA) operated at −3,600 V in negative ionization mode. The vaporizer and ion transfer tube were set at a temperature of 450°C and 350°C, respectively. Full scan measurements (MS1) in the mass range from 450 to 1,100 atomic mass units were collected at a resolution of 120.000. Data processing was based on the package XCMS, version 3.12, running under R, version 4.0.3, for peak recognition and integration (47). Lipid classes were identified based on retention time and molecular species were then matched against an *in silico*-generated lipid database. Mass accuracy of annotated lipids was typically below 2 ppm.

### EM.

For negative staining of OMVs, a protocol was provided by the Cell Microscopy Center (CMC; University Medical Center, Utrecht) (23). In short, copper grids were carbon activated, incubated with 10 μl vesicle solution for 10 to 30 min, and washed three times with PBS. The solution was fixed on the grids using 1% glutaraldehyde (Sigma-Aldrich) in PBS for 10 min and washed two times with PBS and subsequently four times with MQ. The grids were then briefly rinsed with methylcellulose-uranyl acetate (pH 4, provided by the CMC) and incubated for 5 min with methylcellulose-uranyl acetate (pH 4) on ice. Grids were looped out of the solution and air dried. Samples were imaged on a Tecnai-12 electron microscope (FEI, Hillsboro, OR, USA).

### Track dilution assay.

Bacterial killing by HDPs was assessed using track dilution assays, as described before (4). In short, 2 × 10^6^ CFU/ml bacteria was incubated with different concentrations of peptide for 3 h at 37°C in a U-bottom microtiter plate (Corning, New York, USA). For assays with hypervesiculating mutants, supernatant of hypervesiculating and wild-type E. coli was collected by 10-min centrifugation at 4,700 × *g*, filtered over 0.45-μm filters, and used to dilute wild-type bacteria. For OMV protection studies, isolated OMVs of E. coli were added at a final concentration of 500 AU, as defined by the FM4-64 lipid dye. After incubation, the mixture was diluted 2- or 5-fold, of which 10-fold serial dilutions were prepared using medium and 10 μl of each dilution was plated on appropriate agar plates. Plates were incubated at 37°C for 24 h. Minimal bactericidal concentration (MBC) was defined as <200 CFU/ml, the detection limit of this assay.
