# Supplementary figures and images for: Outer Membrane Vesicles Protect Gram-Negative Bacteria against Host Defense Peptides
Source: mSphere. 2021 Jul 7;6(4):e00523-21. doi: 10.1128/mSphere.00523-21 (PMC8386409; doi:10.1128/mSphere.00523-21)

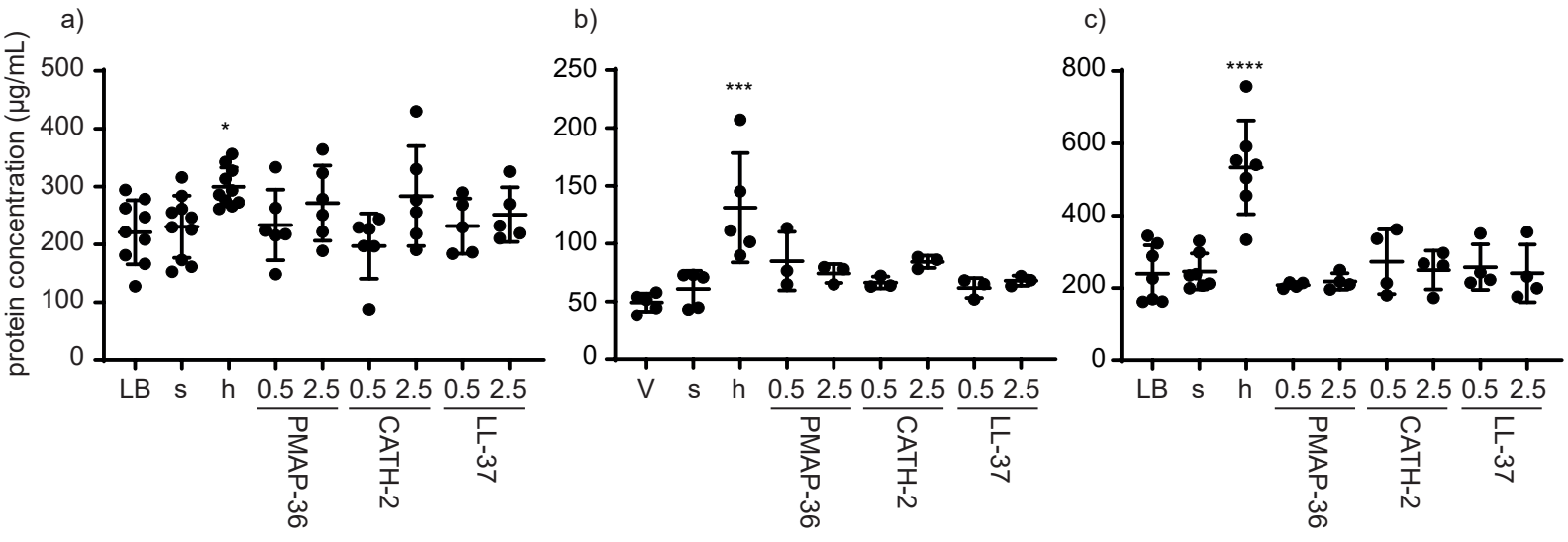

Supplement: FIG S1 [file msphere.00523-21-sf001.pdf]

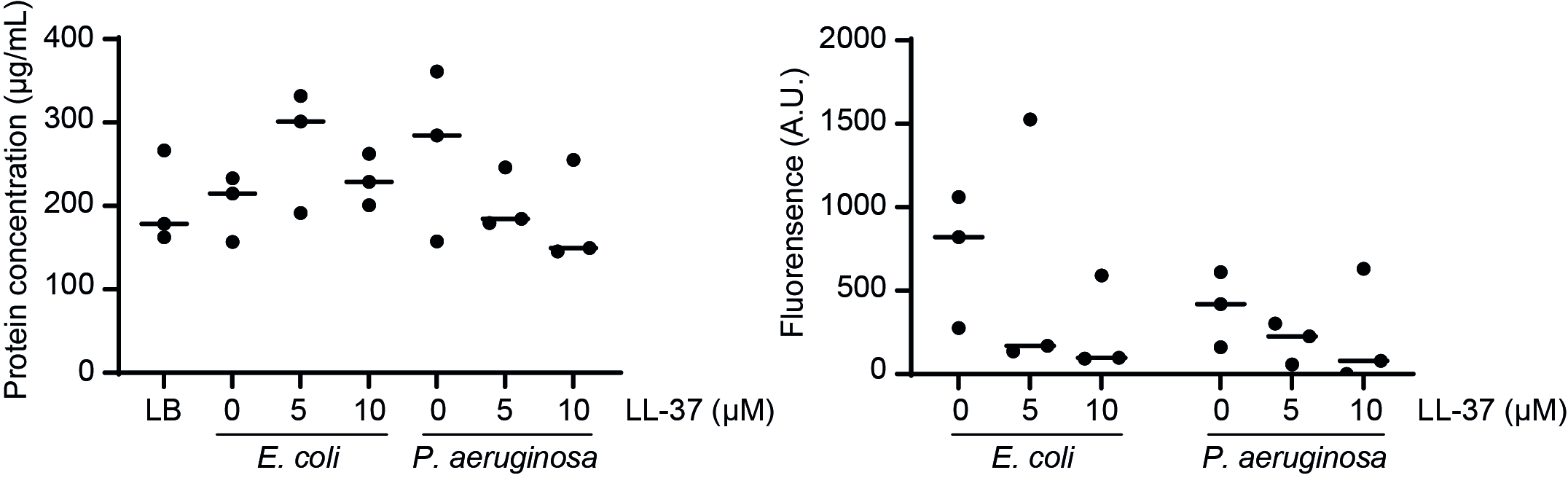

Supplement: FIG S2 [file msphere.00523-21-sf002.tif]

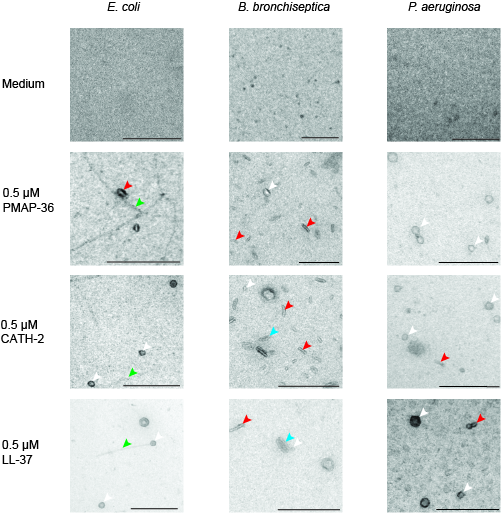

Supplement: FIG S3 [file msphere.00523-21-sf003.tif]

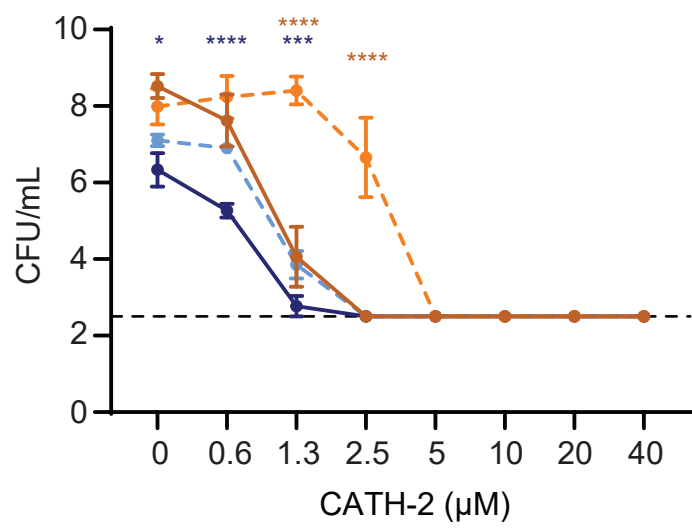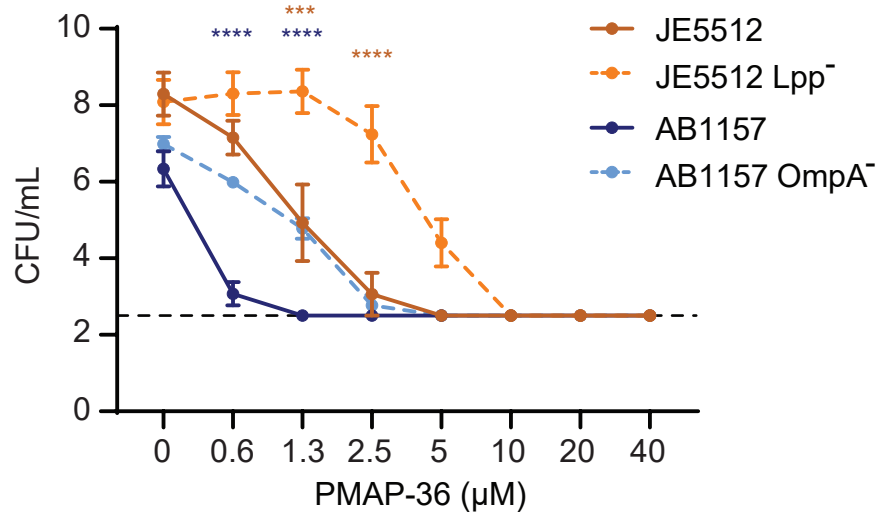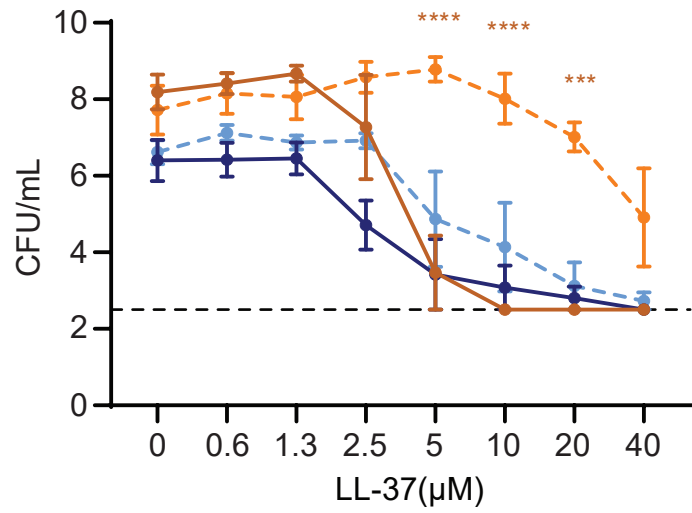

Supplement: FIG S4 [file msphere.00523-21-sf004.pdf]
