# Supplementary material for: Outer Membrane Vesicles Protect Gram-Negative Bacteria against Host Defense Peptides
Source: mSphere. 2021 Jul 7;6(4):e00523-21. doi: 10.1128/mSphere.00523-21 (PMC8386409; doi:10.1128/mSphere.00523-21)
Supplement: Table S1 [file msphere.00523-21-st001.docx]

**Supplementary Table 1: Percentages of lipid species observed in isolated OMVs.** OMVs were isolated from E. coli, B. bronchiseptica and P. aeruginosa, either induced by no treatment (sOMV), heat treatment (hOMVs) or different concentrations of indicated HDPs. aPG = Acyl-phosphatidylglycerol, DLCL = dilyso-cardiolipin, lyso PE = lyso-phosphatidylethanolamine, OL = ornithine lipid, PA = phosphatidic acid, PE = phosphatidylethanolamine, PG = phosphatidylglycerol, PS = phosphatidylserine.

| E. coli | | | | | | | | |  |
| --- | --- | --- | --- | --- | --- | --- | --- | --- | --- |
|  | sOMV | hOMV | 0.5 µM PMAP-36 | 2.5 µM PMAP-36 | 0.5 µM CATH-2 | 2.5 µM CATH-2 | 0.5 µM LL-37 | 2.5 µM LL-37 | |
| aPG | 1,2% | 2,0% | 1,5% | 2,8% | 1,4% | 1,5% | 1,1% | 1,4% | |
| DLCL | 0,3% | 0,2% | 0,3% | 0,3% | 0,4% | 0,5% | 0,2% | 0,3% | |
| lyso PE | 0,8% | 5,8% | 1,5% | 1,0% | 1,5% | 1,1% | 0,5% | 0,6% | |
| OL | 0,0% | 0,0% | 0,0% | 0,0% | 0,1% | 0,0% | 0,0% | 0,2% | |
| PA | 0,6% | 0,3% | 0,7% | 0,8% | 0,7% | 0,9% | 0,5% | 0,5% | |
| PE | 77,9% | 63,5% | 75,6% | 70,1% | 75,7% | 73,1% | 79,7% | 79,4% | |
| PG | 19,1% | 28,0% | 20,3% | 24,8% | 20,3% | 22,7% | 18,0% | 17,5% | |
| PS | 0,1% | 0,2% | 0,1% | 0,1% | 0,1% | 0,1% | 0,0% | 0,1% | |
| B. bronchiseptica | | | | | | | | |  |
|  | sOMV | hOMV | 0.5 µM PMAP-36 | 2.5 µM PMAP-36 | 0.5 µM CATH-2 | 2.5 µM CATH-2 | 0.5 µM LL-37 | 2.5 µM LL-37 | |
| aPG | 0,3% | 0,9% | 0,7% | 1,8% | 0,4% | 0,8% | 0,4% | 1,0% | |
| DLCL | 0,0% | 0,0% | 0,0% | 0,0% | 0,0% | 0,0% | 0,0% | 0,0% | |
| lyso PE | 1,9% | 5,7% | 1,0% | 1,3% | 0,4% | 0,0% | 0,4% | 0,9% | |
| OL | 9,8% | 12,9% | 14,1% | 13,5% | 11,4% | 11,4% | 8,9% | 9,0% | |
| PA | 0,1% | 0,1% | 0,3% | 0,4% | 0,1% | 0,5% | 0,3% | 0,4% | |
| PE | 78,1% | 60,6% | 73,4% | 63,5% | 79,5% | 68,1% | 83,1% | 77,9% | |
| PG | 9,7% | 19,8% | 10,4% | 19,4% | 8,2% | 18,3% | 6,9% | 10,8% | |
| PS | 0,0% | 0,0% | 0,0% | 0,0% | 0,0% | 0,0% | 0,0% | 0,0% | |
| P. aeruginosa | | | | | | | | |  |
|  | sOMV | hOMV | 0.5 µM PMAP-36 | 2.5 µM PMAP-36 | 0.5 µM CATH-2 | 2.5 µM CATH-2 | 0.5 µM LL-37 | 2.5 µM LL-37 | |
| aPG | 2,9% | 5,4% | 3,6% | 4,5% | 2,8% | 3,4% | 1,9% | 0,8% | |
| DLCL | 0,1% | 0,1% | 0,1% | 0,1% | 0,1% | 0,2% | 0,1% | 0,1% | |
| lyso PE | 0,2% | 2,4% | 0,0% | 0,0% | 0,1% | 0,0% | 0,1% | 0,0% | |
| OL | 0,4% | 0,0% | 0,1% | 0,0% | 0,0% | 0,0% | 0,0% | 0,1% | |
| PA | 0,7% | 0,9% | 2,1% | 2,4% | 1,2% | 1,9% | 1,4% | 1,2% | |
| PE | 83,7% | 66,4% | 83,4% | 66,3% | 82,2% | 79,2% | 81,0% | 80,8% | |
| PG | 11,8% | 24,6% | 10,6% | 26,1% | 13,4% | 15,1% | 15,3% | 16,9% | |
| PS | 0,1% | 0,2% | 0,1% | 0,4% | 0,1% | 0,2% | 0,1% | 0,0% | |
